# Hesperidin mitigates cognitive and anxiety-like deficits by enhancing hippocampal antioxidant defenses, reducing neuroinflammation, and preventing neuronal apoptosis in a third-trimester-equivalent rat model of developmental ethanol neurotoxicity

**DOI:** 10.1016/j.ibneur.2026.06.001

**Published:** 2026-06-05

**Authors:** Zhaleh Jamali, Fahimeh Mohseni, Ahmad Salimi, Behzad Garmabi, Mehdi Khaksari

**Affiliations:** a Student Research Committee, School of Medicine, Shahroud University of Medical Sciences, Shahroud, Iran; b Center for Health Related Social and Behavioral Sciences Research, Shahroud University of Medical Sciences, Shahroud, Iran; c Addiction Research Center, Shahroud University of Medical Sciences, Shahroud, Iran; d Social Determinants of Health Research Center, Ardabil University of Medical Sciences, Ardabil, Iran; e Department of Pharmacology and Toxicology, School of Pharmacy, Ardabil University of Medical Sciences, Ardabil, Iran; f Department of Neuroscience, School of Medicine, Shahroud University of Medical Sciences, Shahroud, Iran; g Neuroscience Research Center, Shahroud University of Medical Sciences, Shahroud, Iran

**Keywords:** Ethanol, Fetal Alcohol Spectrum Disorder, Cell death, Oxidative Stress, Neurotoxicity, Hesperidin

## Abstract

Ethanol exposure during brain development has been associated with hippocampal oxidative stress, neuroinflammation, and apoptosis, resulting in lasting cognitive and emotional deficits characteristic of fetal alcohol spectrum disorders (FASD). This study evaluated the neuroprotective potential of Hesperidin, a citrus flavanone with antioxidant and anti-inflammatory actions, against developmental ethanol neurotoxicity. Neonatal Wistar rats received ethanol (5.25 g/kg/day; 11.9% v/v) from postnatal days 2–10 and were subsequently treated intraperitoneally with Hesperidin (25, 50, or 100 mg/kg). Behavioral testing on days 39–45 using the Elevated Plus Maze (EPM) and Morris Water Maze (MWM) showed that ethanol markedly decreased open arm time (Oat%) and entries (OAE%) (P < 0.001, P < 0.01 vs Control), increased escape latency (P < 0.001), and reduced probe target time (P < 0.001 vs Control). Hesperidin at 50 and 100 mg/kg improved all indices (P < 0.05–0.01 vs Ethanol). Ethanol elevated hippocampal MDA and TNF-α (P < 0.001) while decreasing SOD (P < 0.01) and GSH-Px (P < 0.001), Hesperidin normalized these values (P < 0.01–0.001 vs Ethanol). GFAP and cleaved caspase-3 immunoreactivity were also reduced by hesperidin (P < 0.001 vs Ethanol). Overall, Hesperidin afforded dose-dependent neuroprotection by mitigating oxidative stress, inflammation, and apoptosis, thereby improving ethanol-induced behavioral impairments.

## 1 Introduction

Numerous experimental and clinical studies demonstrate that maternal exposure to ethanol presents a significant teratogenic threat (Mohseni et al., 2023). In particular, the ingestion of alcohol during this crucial neurodevelopmental period triggers marked neuroinflammatory reactions and fetal neuronal cell death, thereby interfering with essential neurodevelopmental achievements and resulting in the diverse neurobehavioral and cognitive impairments associated with Fetal Alcohol Spectrum Disorders (FASD) (Kane and Drew, 2021, Moore and Xia, 2022). Although entirely avoidable, FASD continues to represent a pressing global health issue, with alcohol consumption reported in approximately 10% of pregnancies and an estimated prevalence of 7.7 per 1000 individuals (Jacobsen et al., 2022).

Among individuals impacted by FASD, significant and widespread impairments in cognitive function and memory are characteristic and notably prevalent attributes. In this regard, the research conducted by (Mclachlan et al., 2019), revealed that adults diagnosed with FASD display extensive deficits across various cognitive domains; specifically, 93.7% of participants demonstrated cognitive dysfunction, particularly in memory (approximately 43%), alongside a markedly low level of general intellectual functioning (mean IQ ≈ 65). It is imperative to note that the diminished level of intelligence quotient observed in FASD is intricately linked to disruptions in the mechanisms underlying memory consolidation, learning efficacy, and the synthesis of advanced cognitive functions in affected individuals (Mclachlan et al., 2019). Furthermore, a neurocognitive evaluation conducted by Kerdreux et al. in 2024 on children diagnosed with FASD uncovered significant deficits in both working memory and the velocity of memory processing. Within this investigation, out of the 107 children assessed, 28% were classified within the borderline range and 23% within the deficient range concerning overall cognitive performance (Kerdreux et al., 2024). In addition to the widespread cognitive impairments and various memory domains, individuals with FASD exhibit pronounced affective dysregulation, with anxiety disorders surfacing as one of the most prevalent emotional disturbances, impacting approximately 20–45% of those diagnosed with FASD (Wrath et al., 2022).

Preclinical studies utilizing animal models of FASD have similarly revealed analogous impairments in memory and cognitive functioning, accompanied by emotional dysregulations characterized by increased stress and anxiety-like behaviors (Mohseni et al., 2021a, Mohseni et al., 2021b). To establish a connection between these behavioral outcomes and their corresponding neurobiological foundations, animal models subjected to neurodevelopmental ethanol exposure present a distinctive opportunity to investigate FASD-related impairments across various developmental phases, including those that correspond to the human third trimester (Patten et al., 2014).

These models administer ethanol exposure during the initial 10 days postnatally in rodent subjects, which is regarded as comparable to the human third trimester owing to the delayed onset of the brain growth spurt in rodents relative to humans (Patten et al., 2014). This pivotal exposure period precisely aligns with the growth spurt, coinciding with the maturation and differentiation of the hippocampus, a crucial structure for memory and cognitive processes, as well as a region involved in the regulation of emotions within the mesolimbic circuit. Consequently, this model is uniquely capable of capturing the cognitive and anxiety-like impairments associated with hippocampal damage induced by ethanol exposure within this critical developmental window (Goodfellow and Lindquist, 2014, Marengo et al., 2023, Nogales et al., 2025).

Importantly, the developmental stage at which behavioral outcomes are evaluated is also a key factor in interpreting the long-term consequences of early-life ethanol exposure (Subbanna and Basavarajappa, 2022). Adolescence represents an important developmental stage for evaluating the long-term consequences of early-life ethanol exposure. Although neurodevelopmental disturbances may originate during prenatal or early postnatal periods, many behavioral and cognitive abnormalities associated with FASD become more evident later in development as neural systems supporting learning, memory, and emotional regulation are increasingly recruited (Dos Santos et al., 2019, Moore and Xia, 2021). Consequently, adolescence provides a particularly informative window for detecting functional impairments resulting from earlier neurodevelopmental insults and for assessing the potential efficacy of neuroprotective interventions (Dos Santos et al., 2019).

It has been empirically demonstrated that binge-like exposure to ethanol during the third-trimester-equivalent developmental period leads to significant impairments in spatial learning and memory performance, as evidenced by the outcomes of the Morris water maze (MWM) test, in conjunction with heightened anxiety-like behaviors, as indicated by prolonged periods of immobility and diminished distances traversed in the open field test in mice assessed at postnatal day 60 (PND 60). These documented behavioral deficits are associated with an imbalance between excitatory and inhibitory synaptic activity within the hippocampus, accompanied by marked neuroapoptosis (Nogales et al., 2025). Additionally, another study has demonstrated that ethanol exposure during the third-trimester-equivalent period is linked to anxiety-related behaviors in the elevated plus maze in 39-day-old Wistar rats, with concurrent reductions in GFAP expression, an established astrogliosis marker, and a mitigation of alcohol-induced necrotic cell death associated with neurotoxicity (Rafaiee and Mohseni, 2025).

Furthermore, additional studies have reported that ethanol exposure induces neurotoxicity in the developing hippocampus of Wistar rats during the third-trimester-equivalent period, mediated through enhanced oxidative stress, increased inflammatory responses, and apoptotic cell death, ultimately culminating in substantial spatial memory deficits in 40-day-old rats, as evaluated in the MWM test (Jafari et al., 2021). While the deleterious effects of ethanol and the consequent modifications in the hippocampus, including oxidative stress, increased astrogliosis, and neuroapoptosis, are thoroughly documented, certain investigations suggest that antioxidant interventions may mitigate these repercussions (Król et al., 2024, Navarro-Cruz et al., 2024). Specifically, antioxidants originating from natural sources, due to their inherent antioxidant characteristics, constitute compounds with considerable neuroprotective efficacy against the neurotoxic consequences of ethanol (Gasparyan et al., 2023, Mohseni et al., 2020a, Navarro-Cruz et al., 2024). Hesperidin, a bioflavonoid that is intrinsically prevalent in citrus fruits such as oranges, lemons, and grapefruits, demonstrates significant antioxidant properties and is regarded as one of the foremost natural compounds that facilitate cellular defense mechanisms against oxidative stress (Hajialyani et al., 2019, Pal et al., 2024, Subhan and Siddique, 2024).

It is noteworthy that contemporary experimental findings emphasize that the remarkable antioxidant properties of Hesperidin are fundamental to its neuroprotective functions, particularly in pathological situations where oxidative stress compromises neuronal equilibrium and instigates pro-inflammatory signaling pathways such as NF-κB activation (Parhiz et al., 2015). By inhibiting these pathways, Hesperidin effectively mitigates the release of pro-inflammatory cytokines, including TNF-α and IL−1β, thereby disrupting the subsequent apoptotic cascades that result in neuronal cell demise (Parhiz et al., 2015, Tejada et al., 2018). Despite the growing body of evidence supporting the neuroprotective properties of Hesperidin, its capacity to counteract ethanol-induced oxidative stress, particularly its impact on hippocampal neuroinflammation, neuronal apoptosis, and the associated behavioral impairments stemming from hippocampal dysfunction, has not yet been investigated in FASD models. Therefore, the present study sought to determine whether Hesperidin could restore hippocampal antioxidant defenses, attenuate neuroinflammatory activity reflected by TNF-α and GFAP expression, and reduce neuronal apoptosis, thereby improving hippocampus-dependent learning, memory, and anxiety-related behaviors following third-trimester equivalent ethanol exposure in Wistar rats. To address these objectives, we employed validated behavioral assays, including the MWM to evaluate spatial learning and memory and the EPM to assess anxiety-like responses. By integrating behavioral, biochemical, and molecular measures, this study aims to provide new insights into the therapeutic potential of Hesperidin as a neuroprotective compound capable of mitigating the progression of ethanol-induced neurodevelopmental impairments.

## 2 Materials and methods

### 2.1 Animals

**Fig. 1:**
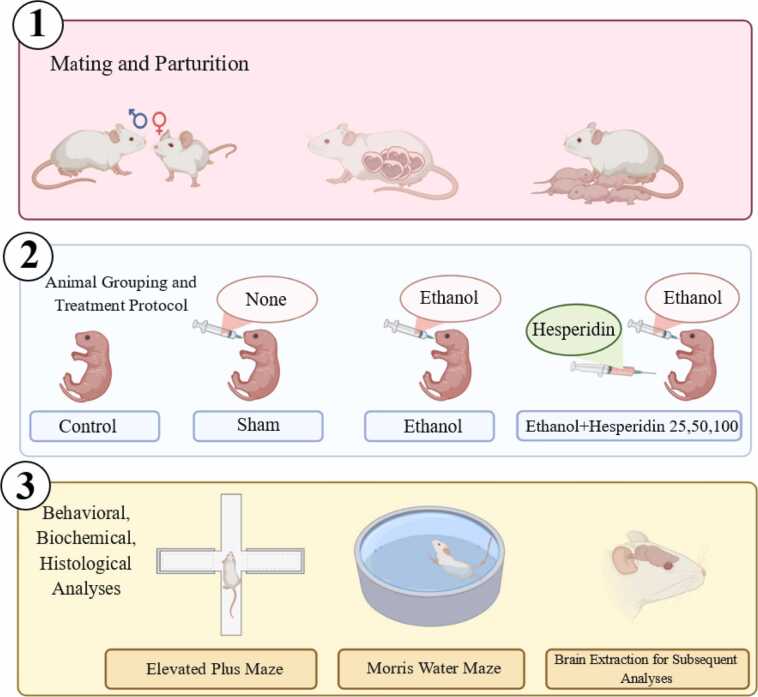
Schematic representation of the experimental timeline and methodological framework employed in the animal study (Created with BioRender.com). (1) Mating and parturition: Adult Wistar rats were paired during the estrus phase, with parturition occurring on PD0, (2) Animal grouping and treatment protocol: On PD2, male pups were allocated into Control, Sham, Ethanol, and Ethanol + Hesperidin (25, 50, 100 mg/kg) groups. Ethanol was administered orally in a milk solution from PD2 to PD10, followed by intraperitoneal hesperidin in graded doses, (3) Behavioral, biochemical, and histological assessments: EPM was conducted on PD40, MWM training and probe trials on PD41–45, followed by hippocampal tissue collection for biochemical assays (SOD, GSH‑Px, MDA, TNF‑α) and histological evaluations (GFAP, cleaved caspase‑3).

A diagrammatic depiction of the experimental framework is illustrated in Fig. 1.

Adult virgin Wistar rats (40 females and 40 males; 250–280 g) were obtained from the Animal Centre of the Faculty of Medicine, Shahroud University of Medical Sciences. Females and males were housed together for mating under standard laboratory conditions. The offspring were delivered within a timeframe of 21–23 days following copulation, with the day of parturition designated as day zero. On the second postnatal day (PD2), a systematic allocation of 60 male pups, each weighing between 7 and 9.5 g, was executed into six distinct groups, with each group consisting of 10 individuals. Interventions were administered within a timeframe corresponding to the third trimester of human gestation, specifically from PD2 to PD10.

Group 1 – Control: In this cohort, no interventions were performed. The progeny did not receive any form of oral or injectable treatment throughout the duration of the study.

Group 2 – Sham: The neonates in this group underwent the insertion of a gavage needle into the esophagus for a duration ranging from 4 to 8 s, without any fluid administration, thereby replicating the physical manipulation typically associated with oral gavage.

Group 3 – Ethanol: This cohort was subjected to the administration of ethanol solubilized in a milk solution through oral gavage.

Group 4, 5, 6 – Ethanol + Hesperidin: The subjects in these groups received ethanol in a milk solution via oral gavage, which was subsequently followed by an intraperitoneal administration of hesperidin at concentrations of 25, 50, and 100 mg/kg.

From PD2 to PD10, the offspring pups in the ethanol treatment group underwent intragastric intubation utilizing a straight stainless steel gavage needle, which was saturated with corn oil (length: 2.54 cm, ball diameter: 1.25 mm, Cadence Science®). Consequently, the offspring pups in the ethanol group were administered a cumulative daily dose of ethanol (5.25 g/kg, 11.9% v/v) in a milk solution (27.8 ml/kg), which was divided into two separate feedings administered bi-daily at 120-minute intervals (Rafaiee et al., 2025). Exposure to neonatal alcohol has been associated with an increased latency in the initiation of milk suckling, a reduction in suckling duration, and a decrease in overall milk intake; these factors collectively contribute to a lower weight in comparison to control subjects of equivalent age. To alleviate these nutrition-related deficiencies, a supplementary dose of ethanol-free milk solution (exclusively milk feeding) was provided 120 min following the administration of the second ethanol dose. Immediately after the third oral gavage, Hesperidin was subsequently administered intraperitoneally at graded doses of 25, 50, and 100 mg/kg (Huang et al., 2020). The third gavage (ethanol free milk) was applied only to pups in the ethanol and ethanol + hesperidin groups (Groups 3–6). Sham pups underwent the same number of daily gavage insertions without fluid to control for procedure related stress, whereas control pups did not receive any gavage. To accurately emulate rat breast milk, a specialized milk solution was meticulously crafted in the laboratory of Shahroud University of Medical Sciences, adhering to a previously established protocol (West et al., 1984). All subjects across experimental groups, inclusive of the control group, underwent daily weight assessments from postnatal day two until postnatal day ten. For the purposes of intervention and/or weighing, the litters were systematically extracted from their dams and placed upon a heating pad within containers designed to provide a suitable thermal environment for the neonates during this critical phase of development. Following each separation event, the litters were swiftly returned to the maternal enclosure. Until the weaning process commenced on the twenty-fifth day post-partum, the dams were housed within the same environment as their offspring. Subsequently, the mothers were individually accommodated apart from their litters. Behavioral evaluations were conducted on postnatal day 39–45 in Wistar rats, which corresponds to a developmental stage that parallels adolescence and the onset of puberty in human subjects. All experimental procedures were conducted in accordance with the ethical standards for animal experimentation and were approved by the Ethics Committee of Shahroud University of Medical Sciences under the code IR.SHMU.REC.1401.082.

### 2.2 Elevated plus maze (EPM) test

The EPM assessment was conducted 39 days after the birth of the pups to evaluate anxiety‑like behaviors in rats. The apparatus, initially delineated by Handley and Mithani, comprised a plus-shaped maze featuring four arms arranged at orthogonal angles around a central platform. Two open arms (25 × 5 × 5 cm) were situated in opposition to one another and perpendicular to two enclosed arms (50 × 10 × 40 cm), which were bordered by 40 cm-high opaque barriers. The arms radiated from a central platform (10 × 10 cm) elevated 50 cm above the ground. Before the commencement of testing, animals were provided with a 60-minute habituation period in the testing room to allow adequate acclimation to the environment. Each rat was carefully positioned on the central platform facing an open arm and permitted to explore autonomously for 5 min under regulated light and auditory conditions. The entirety of the session was captured utilizing a computerized video-tracking system. An entry was defined as the placement of all four paws within an arm. To ascertain the percentage of time allocated to the open arms (%OAT) and the percentage of entries into the open arms (%OAE), the subsequent mathematical equations were employed.

$$\%\mathrm{OAT}=\frac{100\times \mathrm{OAT}}{\mathrm{OAT}+\mathrm{CAT}}$$

$$\%\mathrm{OAE}=\frac{100\times \mathrm{OAE}}{\mathrm{OAE}+\mathrm{CAE}}$$

In these equations, OAT signifies the duration (measured in seconds) that the subject animal occupied the open arms, CAT indicates the duration spent in the closed arms, OAE pertains to the frequency of entries into the open arms, and CAE represents the frequency of entries into the closed arms. By utilizing these variables, the ratio of time and the frequency of entries pertaining to the open arms were systematically quantified, which function as valid indicators of anxiety-like behavior in rodent models (Carobrez and Bertoglio, 2005).

### 2.3 Morris water maze (MWM) task

The evaluation of hippocampus-dependent memory and spatial learning occurred 40 days postnatally utilizing the MWM task. The subjects underwent a training regimen spanning four consecutive days, comprising four trials with intertrial intervals of 30 s, aimed at locating a submerged platform. The apparatus was systematically divided into four quadrants (Northwest, Northeast, Southwest, and Southeast), with the platform strategically positioned in the Northwest quadrant, while the subjects were randomly and gently introduced into one of the quadrants facing the perimeter of the pool. The subjects were allocated a designated duration to locate and ascend the concealed platform, permitted to remain on the platform for a duration of 30 s. Should the rodents fail to locate the platform within a 1-minute timeframe, an experimenter would assist them in accessing the platform. Multiple parameters including escape latency, total distance traversed, and velocity were meticulously recorded through an automated video tracking system. Subsequently, an average was computed for the four daily trials. A probe test was conducted to assess spatial memory on the 45th day postnatally (1 day subsequent to the final training trial). For this assessment, the platform was removed from the tank, allowing each rat to swim freely. Thereafter, the duration spent in the quadrant designated for the platform (target quadrant) was compared to that spent in the other three quadrants, an increased duration in the target quadrant signified enhanced memory retention (Carobrez and Bertoglio, 2005, Morris, 2008).

### 2.4 Tissue allocation for biochemical and histological analyses

After completion of the behavioral tests, all animals were deeply anesthetized with ketamine/xylazine, 80/10 mg/kg, i.p. and sacrificed by decapitation using an automated guillotine (Rafaiee et al., 2024). The brains were rapidly removed and processed for subsequent biochemical and histological analyses. To enable parallel assessment of biochemical and histological outcomes while minimizing animal use, brain tissue from all animals (n = 10 per group) was systematically divided between the two types of analyses. Each experimental group comprised 10 animals, and the same animals contributed tissue for both biochemical and histological evaluations. Following brain removal, the left and right hemispheres were separated, one hemisphere was used for fresh hippocampal dissection and subsequent biochemical assays, while the contralateral hemisphere was fixed in 4% paraformaldehyde and processed for histological examination.

### 2.5 Biochemical assessment

For biochemical analyses, fresh hippocampal tissue was obtained from one hemisphere of each brain as described in Section 2.4. The dissected hippocampi were immediately placed on ice and homogenized in cold RIPA buffer supplemented with a protease inhibitor cocktail. The homogenates were then centrifuged at 3000*g* for 20 min at 4 °C, and the resulting supernatants were collected for subsequent enzymatic and oxidative stress measurements (Sabzali et al., 2022).

### 2.6 Malondialdehyde (MDA) measurement

MDA levels were quantified following the protocol provided by the MDA assay kit (ZellBio, Germany). After the homogenization of hippocampal samples in a 1.15% potassium chloride (KCl) solution within a two-minute timeframe, MDA concentration was assessed using the TBARS methodology. The homogenized samples were subjected to heating (100°C) alongside a combination of thiobarbituric acid (10%, 2 ml) and trichloroacetic acid (10%, 1 ml) for a duration of one hour. Subsequent to cooling and centrifugation of the resultant solution at ambient temperature, the precipitates were discarded. The resulting supernatant was subsequently transferred to a microplate. The absorbance of the reaction mixture was measured at 535 nm utilizing a microplate reader (ELx800, BioTek, USA). MDA concentrations were established by analyzing peak areas in conjunction with standard curves (Farhadi et al., 2022).

### 2.7 Measurement of glutathione peroxidase (GSH-Px), superoxide dismutase (SOD)

The concentrations of SOD, and GSH-Px were quantified in accordance with the protocols established by the kit manufacturer (ZellBio, Germany). Following the processes of homogenization and centrifugation of the hippocampal specimens through the incorporation of a PBS solution (100 mM, pH 7.4), the pink-colored supernatants were extracted. Subsequent to the interaction between the chromogenic reagent and the enzymes SOD and GSH-Px, the absorbance was recorded at wavelengths of 412 and 420 nm, respectively, utilizing a microplate reader (ELx800, BioTek, USA) (Mohseni et al., 2020b).

### 2.8 Tumor necrosis factor-α (TNF-α) measurements

The levels of TNF-α were assessed following the guidelines provided by the kit (Diaclone). The wells of the microtiter plates were coated with monoclonal antibodies specifically targeting TNF-α. Samples with both unknown and known concentrations of TNF-α were introduced into the wells. Concurrently, the TNF-α antigen was allowed to incubate with a biotinylated monoclonal antibody. Subsequently, streptavidin horseradish peroxidase (HRP) was administered to the wells. After the removal of unbound enzymes, the bound enzyme was combined with a substrate solution to yield a colored solution. It is important to note that the intensity of the resulting product was directly proportional to the concentration of TNF-α present. The final values were expressed as picograms per milligram of total protein (Farhadi et al., 2022).

### 2.9 Tissue preparation

For histological evaluation, the contralateral cerebral hemisphere from each animal, as described in Section 2.4, was immediately immersed in 4% paraformaldehyde prepared in 0.1 M phosphate‑buffered saline. Subsequently, the fixed cerebral tissues were processed, embedded in paraffin wax, and post‑fixed for three days. In accordance with the Paxinos atlas, the paraffin‑embedded brain specimens were coronally sectioned at 5 µm thickness using a microtome and subjected to various staining techniques (Mohseni et al., 2020b).

### 2.10 Immunohistochemical and immunofluorescence staining

For the quantification of cleaved caspase−3 and GFAP expression, immunofluorescence and immunohistochemical staining were executed employing specific antibodies. Following a 30-minute incubation at 60 °C, the paraffin was extracted and subsequently cleared using xylene. A gradient of ethanol was applied to achieve rehydration, and to inhibit endogenous peroxidase activity, a solution of 10% H2O2 in methanol was utilized for ten minutes; the sections were then rinsed with Tris buffer, after which antigen retrieval was performed in citrate buffer (C6H5Na3O7•2H2O; pH = 6) via autoclaving for 11 min. The samples were subjected to blocking with 1% fetal bovine serum in 0.3% Triton X−100 post washing in PBS, followed by incubation with a primary antibody (Abcam, UK) at 4 °C overnight; the optimal dilution ratio was determined to be 1/100 (Farhadi et al. 2022). The horseradish peroxidase (HRP) conjugated secondary antibody (GFAP) was incubated for 30 min at ambient temperature after the introduction of 3,3′-diaminobenzidine (DAB) for antigen detection. Subsequently, counterstaining was executed with hematoxylin (Sigma) to facilitate visualization under a microscope. Furthermore, for the detection of cleaved caspase−3, the sections were treated with an anti-rabbit secondary antibody for one hour in a dark environment, conjugated with a fluorochrome (Abcam, UK). Following this, the sections were counterstained with 4′,6 diamidino−2-phenylindole (DAPI) for five minutes to label the nucleus. Subsequent to a washing procedure, the fluorescence signals were analyzed using a fluorescence microscope. The quantification of GFAP and cleaved caspase−3 positive cells was performed (blindly) within the right hippocampal CA1 region of each slide at 400 × magnification. Images were analyzed utilizing ImageTool 2 software. The total cell count was conducted along a 400 μm transect (0.160 mm2) of the CA1 region of the right hippocampus. A slide processed without a primary antibody served as the negative control (Farhadi et al., 2022).

### 2.11 Data analysis

Data analysis was performed using Graph Pad Prism 9. A one-way ANOVA was employed to determine the differences among the animal groups, with Tukey’s post hoc tests following. To assess differences between groups at each time point, a two-way repeated measures ANOVA with Bonferroni’s multiple comparisons test was applied. The results were presented as mean ± SEM, with P values of ≤ 0.05 considered statistically significant.

## 3 Results

### 3.1 Hesperidin mitigated developmental ethanol-induced anxiety-like behaviors

**Fig. 2:**
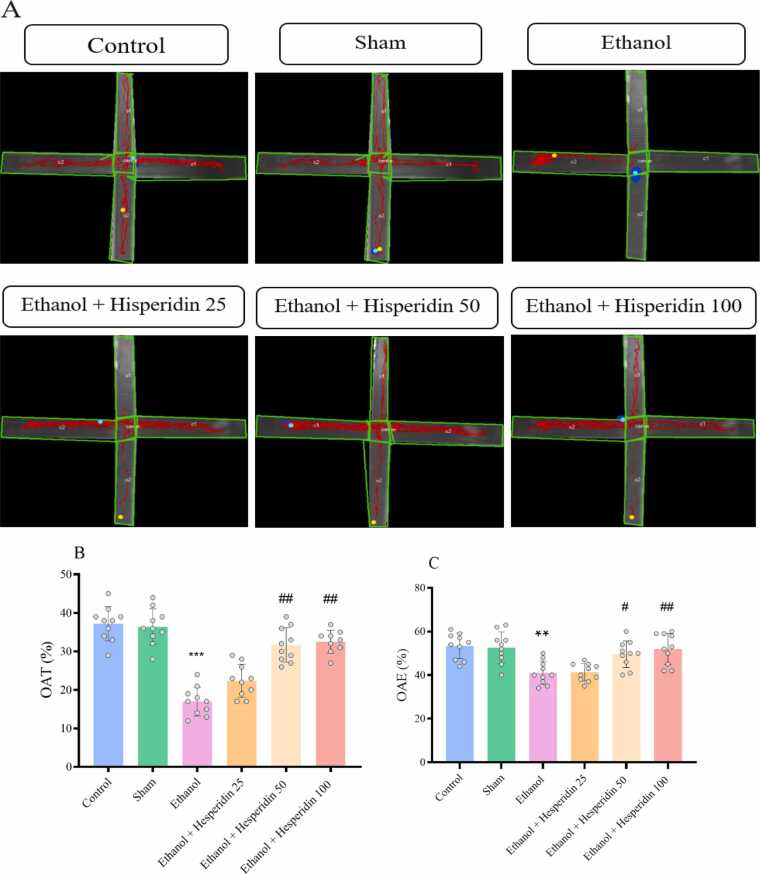
The impact of Hesperidin on anxiety-like behaviors elicited by ethanol, as evaluated using the Elevated Plus Maze (EPM) assay. (A) Exemplary video-tracking diagrams illustrating the movement trajectories (red traces). In each illustration, the vertical axis represents the open arms, whereas the horizontal axis denotes the closed arms. (B) Illustrates the proportion of time spent in the open arms (Oat%) and (C) Illustrates the proportion of entries into the open arms (OAE %). ^***^ vs Control group (P < 0.001). ^**^ vs Control group (P < 0.01). ^##^ vs Ethanol group (P ^<^ 0.01). ^#^ vs Ethanol group (P < 0.05).

To assess anxiety‑like behavior, the percentage of open‑arm time (Oat%) and open‑arm entries (OAE%) in the elevated plus maze were analyzed using one‑way ANOVA. A significant main effect of treatment was observed for both Oat% (F (5,54) = 34.82, p < 0.0001) and OAE% (F (5,54) = 27.46, p < 0.0001). Post‑hoc Tukey’s test revealed that developmental ethanol exposure markedly reduced both Oat% and OAE% compared with the control and sham groups (p < 0.001 for Oat%; p < 0.01 for OAE%), indicating increased anxiety‑like behavior. Hesperidin at 25 mg/kg did not significantly improve either parameter compared with the ethanol group (p > 0.05), suggesting a lack of anxiolytic effect at this dose. In contrast, Hesperidin at 50 mg/kg and 100 mg/kg significantly elevated Oat% (p < 0.01) and increased OAE% (p < 0.05 and p < 0.01, respectively), demonstrating a dose‑dependent anxiolytic effect against ethanol‑induced behavioral deficits (Fig. 2).

### 3.2 Hesperidin mitigates developmental ethanol‑induced impairments in spatial learning and memory

**Fig. 3:**
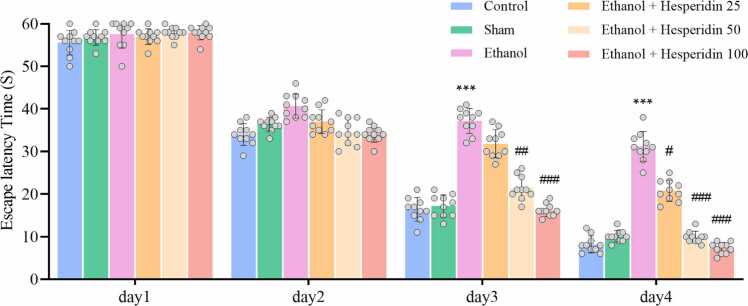
Escape latency duration to reach the concealed platform throughout four consecutive trial days in the Morris water maze task across experimental groups. ^***^ vs Control group (P < 0.001). ^###^ vs Ethanol group (P < 0.001). ^##^ vs Ethanol group (P ^<^ 0.01). ^#^ vs Ethanol group (P < 0.05).

In the MWM test, data concerning escape latency were analyzed using a two-way repeated-measures ANOVA, with treatment group and training day as independent variables. The analysis revealed significant main effects of group (F (5216) = 188.4, p < 0.0001) and time (F (3216) = 356.1, p < 0.0001). Furthermore, a significant interaction between group and time was observed (F (15,216) = 38.72, p < 0.0001). Following the significant interaction, Bonferroni’s post hoc analysis was conducted to determine specific differences. The results indicated that the ethanol-exposed group exhibited significantly longer escape latencies compared to the control group on days 3 and 4 (p < 0.001). In contrast, treatment with Hesperidin at 50 mg/kg (p < 0.01) and 100 mg/kg (p < 0.001) significantly reduced escape latency relative to the ethanol group, whereas the 25 mg/kg dose did not produce a statistically significant difference (p > 0.05) (Fig. 3).

**Fig. 4:**
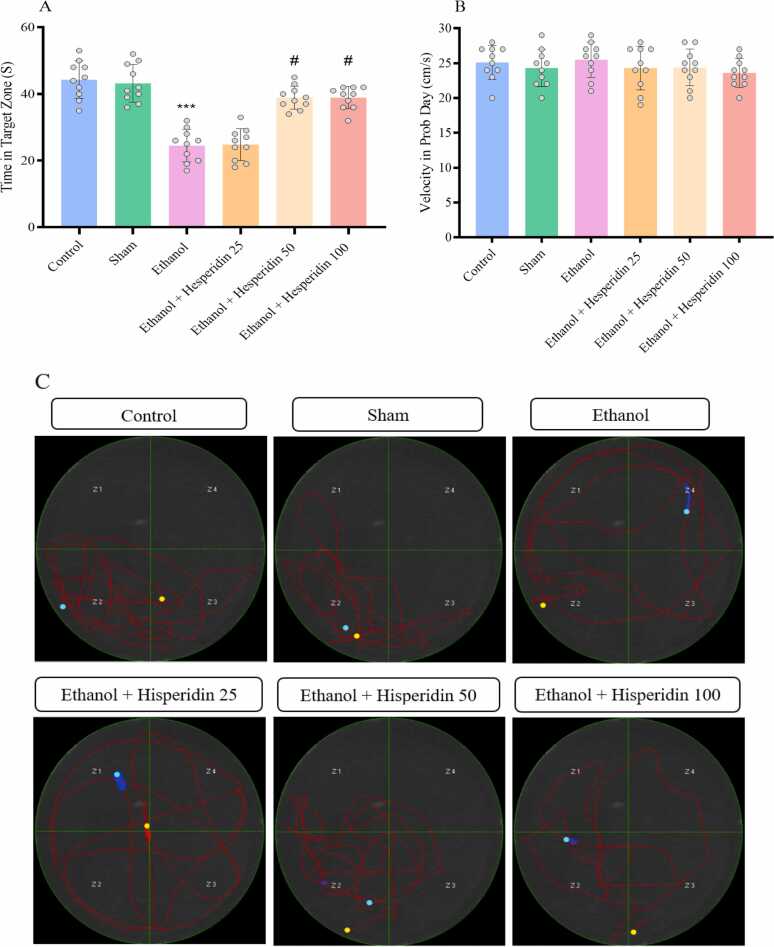
The amount of time designated within the specified target region (A), the swimming velocity recorded on the day of the probe (B), and illustrations of swimming paths observed during the probe trial (day 5) of the Morris water maze task across various experimental groups (C). *** vs Control group (P < 0.001). # vs Ethanol group (P < 0.05).

In the probe trial, one-way ANOVA followed by Tukey’s post hoc test demonstrated a significant difference among groups (F (5,54) = 35.41, p < 0.0001). Ethanol exposure markedly decreased the time spent in the target zone compared to controls (p < 0.001), while Hesperidin at 50 and 100 mg/kg significantly increased target zone occupancy relative to the ethanol group (p < 0.05), with no significant effect observed at 25 mg/kg (p > 0.05). For swimming velocity on the probe day, one-way ANOVA showed no significant differences among groups (F (5,54) = 0.59, p = 0.67), indicating that the observed changes in spatial memory were not attributable to alterations in locomotor activity (Fig. 4).

### 3.3 Hesperidin enhances the ethanol-induced reductions in hippocampal SOD and GSH-Px levels

**Fig. 5:**
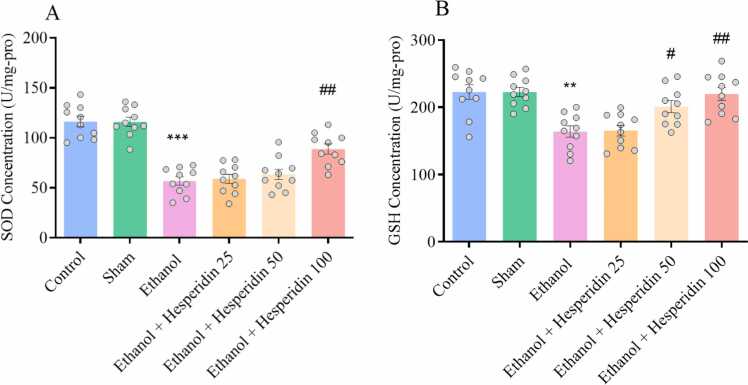
The impact of Hesperidin on hippocampal SOD (A) and GSH‑Px (B) levels following developmental ethanol exposure. *** vs Control group (P < 0.001). ** vs Control group (P < 0.01). ## vs Ethanol group (P < 0.01). # vs Ethanol group (P < 0.05).

One-way ANOVA revealed a significant overall effect of treatment on SOD concentration (F (5,54) = 9.12, p < 0.0001). Tukey’s post-hoc test showed that SOD levels were significantly diminished in rats subjected to ethanol treatment compared with the control group (p < 0.001). Administration of Hesperidin at 25 mg/kg and 50 mg/kg did not produce significant alterations relative to ethanol (p > 0.05), whereas the 100 mg/kg dosage resulted in a significant restoration of SOD activity (p < 0.01). Similarly, the analysis of GSH-Px demonstrated a significant main effect of treatment (F (5,54) = 7.45, p < 0.0001). Tukey’s post-hoc comparisons indicated that ethanol exposure led to a marked decrease in hippocampal GSH-Px concentration compared with controls (p < 0.01). Hesperidin at 25 mg/kg did not produce significant effects (p > 0.05), while the 50 mg/kg dose resulted in a modest but statistically significant increase (p < 0.05). The 100 mg/kg dosage elicited a more substantial recovery of GSH-Px levels (p < 0.01) (Fig. 5).

### 3.4 Hesperidin attenuates developmental ethanol-induced increases in hippocampal MDA and TNF-α levels

**Fig. 6:**
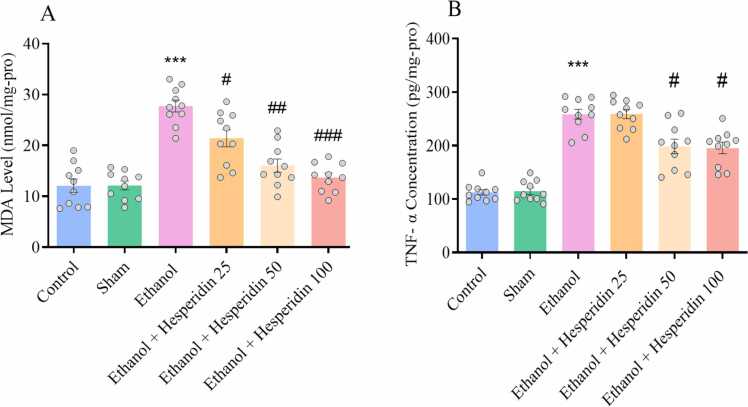
The impact of Hesperidin on hippocampal MDA (A) and TNF-α (B) levels following developmental ethanol exposure. ^***^ vs Control group (P < 0.001). ^###^ vs Ethanol group (P < 0.001). ^##^ vs Ethanol group (P ^<^ 0.01). ^#^ vs Ethanol group (P < 0.05).

One‑way ANOVA revealed a significant overall effect of treatment on hippocampal MDA levels (F (5,54) = 12.47, p < 0.0001). Tukey’s post‑hoc comparisons showed that MDA levels were significantly elevated in the ethanol‑treated group compared with the control group (p < 0.001). Treatment with Hesperidin produced dose‑dependent reductions in this lipid peroxidation marker, with a modest decrease observed at 25 mg/kg (p < 0.05), a greater reduction at 50 mg/kg (p < 0.01), and a pronounced normalization at 100 mg/kg (p < 0.001) compared with the ethanol group. Similarly, analysis of TNF‑α concentrations demonstrated a significant main effect of treatment (one‑way ANOVA: F (5,54) = 9.63, p < 0.0001). Tukey’s post‑hoc test indicated that ethanol exposure significantly increased TNF‑α levels relative to the control group (p < 0.001), reflecting enhanced neuroinflammatory responses. Administration of Hesperidin at 25 mg/kg did not significantly alter TNF‑α levels compared with the ethanol group (p > 0.05), whereas treatment with Hesperidin at 50 mg/kg and 100 mg/kg significantly reduced TNF‑α concentrations relative to the ethanol group (p < 0.05), indicating an attenuation of ethanol‑induced neuroinflammation (Fig. 6).

### 3.5 Hesperidin attenuates developmental ethanol-induced hippocampal astrogliosis

**Fig. 7:**
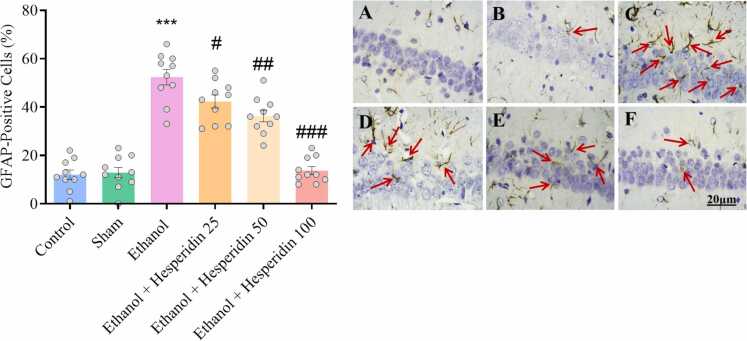
The impact of Hesperidin on hippocampal GFAP- positive cells following developmental ethanol exposure. ^***^ vs Control group (P < 0.001). ^###^ vs Ethanol group (P < 0.001). ^##^ vs Ethanol group (P ^<^ 0.01). ^#^ vs Ethanol group (P < 0.05).

One‑way ANOVA revealed a significant effect of treatment on the percentage of GFAP‑positive cells in the hippocampal CA1 region (F (5,54) = 18.26, p < 0.0001). Tukey’s post‑hoc analysis demonstrated that ethanol exposure significantly increased the proportion of GFAP‑positive cells compared with the control group (p < 0.001), indicating marked reactive astrogliosis. Administration of Hesperidin significantly attenuated this ethanol‑induced astroglial activation in a dose‑dependent manner. Specifically, treatment with 25 mg/kg Hesperidin produced a modest but significant reduction in GFAP‑positive cells compared with the ethanol group (p < 0.05), while 50 mg/kg resulted in a greater decrease (p < 0.01). The highest dose of Hesperidin (100 mg/kg) produced the most pronounced attenuation (p < 0.001), restoring GFAP expression toward levels observed in control animals (Fig. 7).

### 3.6 Hesperidin reduces developmental ethanol-induced hippocampal apoptosis

**Fig. 8:**
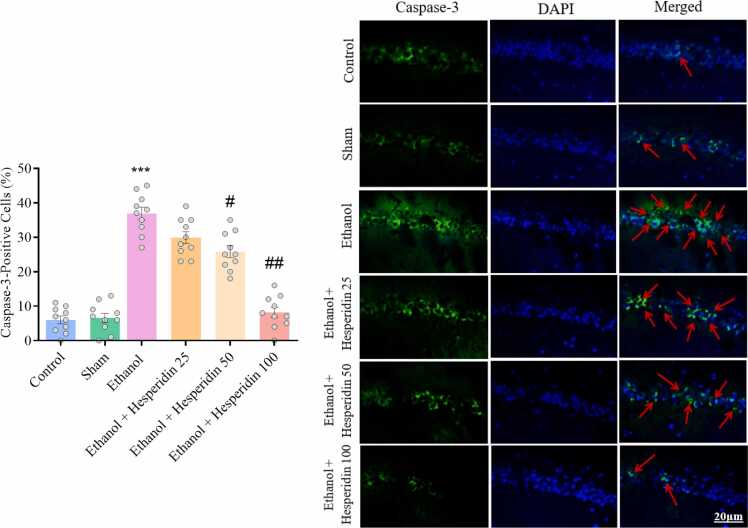
The impact of Hesperidin on hippocampal caspase−3 positive cells following developmental ethanol exposure. ^***^ vs Control group (P < 0.001). ^##^ vs Ethanol group (P < 0.01). ^#^ vs Ethanol group (P < 0.05).

One‑way ANOVA revealed a significant overall effect of treatment on the percentage of caspase‑3‑positive cells in the hippocampal CA1 region (F (5,54) = 15.72, p < 0.0001). Tukey’s post‑hoc analysis demonstrated that ethanol exposure markedly increased apoptotic cell counts compared with the control group (p < 0.001), indicating robust ethanol‑induced hippocampal apoptosis. Administration of Hesperidin showed a dose‑dependent protective effect. The 25 mg/kg dose did not significantly alter caspase‑3‑positive cell levels compared with the ethanol group (p > 0.05), whereas 50 mg/kg produced a significant reduction (p < 0.05). The highest dose, 100 mg/kg, elicited the most substantial anti‑apoptotic effect relative to ethanol (p < 0.01), bringing apoptotic cell levels closer to those observed in control animals (Fig. 8).

## 4 Discussion

The present study demonstrates that Hesperidin exerts significant neuroprotective effects in a rat model of FASD representing the third trimester. These effects appear to arise mainly through the modulation of key molecular and behavioral parameters. Specifically, Hesperidin shows potent antioxidant and neuroprotective actions by enhancing GSH-Px and SOD activities while reducing MDA levels. It also attenuates neuroinflammatory mediators such as TNF-α and GFAP and suppresses neuroapoptosis, as indicated by decreased caspase−3 expression in the hippocampal CA1 region.

The normalization of these neurobiochemical hallmarks was accompanied by marked improvements in hippocampus‑dependent learning, memory, and affective behaviors, reflected by enhanced performance in the MWM and EPM tests. EPM analysis showed that Hesperidin significantly increased both OAE% and Oat% compared to the ethanol-only group. Similarly, Hesperidin enhanced MWM performance by reducing escape latency during acquisition and increasing time spent in the target zone during the probe phase. Consistent with previous reports, the anxiolytic and cognitive‑enhancing effects of Hesperidin have been demonstrated in several independent studies. In a rat model of unpredictable chronic mild stress (UCMS), Hesperidin administration significantly improved cognitive performance and reduced anxiety‑like behaviors. These behavioral improvements were accompanied by normalization of serum corticosterone levels, a key biomarker of chronic stress. Molecular analyses further revealed enhanced antioxidant defenses, reflected by increased SOD and CAT activities, reduced MDA levels, upregulated BDNF expression, and decreased apoptosis in the hippocampal CA1 region (Ahmed et al., 2022). Similarly, the aglycone form of Hesperidin (Hesperetin) showed comparable anxiolytic and cognitive-enhancing effects in a rat model of Alzheimer’s disease. These improvements were linked to increased activities of SOD, CAT, and glutathione reductase (GRx), alongside significant reductions in MDA levels (Hajizadeh Moghaddam et al., 2020). Collectively, this evidence suggests that Hesperidin’s antioxidant properties confer both anxiolytic and memory-enhancing effects, supporting our current findings. By restoring redox homeostasis and inhibiting neuronal apoptosis, Hesperidin protects against oxidative stress-mediated neurotoxicity. These mechanisms ultimately lead to improved learning and memory performance (Bansal et al., 2024, Justin Thenmozhi et al., 2017). Consequently, the modulation of oxidative stress pathways and apoptosis inhibition may represent the central mechanistic axis underlying Hesperidin’s restorative effects (Bansal et al., 2024).

Hesperidin exerts antioxidant effects through multiple complementary mechanisms involving both direct and indirect pathways. It directly scavenges free radicals and reactive oxygen species (ROS), including superoxide and hydroxyl radicals (Pal et al., 2024). This activity reflects the classical bioflavonoid redox mechanism, whereby hydrogen atoms or electrons are donated to unstable radicals, thereby terminating chain reactions. Through this mechanism, Hesperidin reduces lipid peroxidation biomarkers such as MDA and myeloperoxidase (MPO) (Pal et al., 2024, Pyrzynska, 2022). In addition, it upregulates Nrf−2, a key regulator of the antioxidant response that mitigates oxidative stress–induced damage (Elavarasan et al., 2012). Activation of this pathway enhances the expression and activity of antioxidant enzymes, including HO−1, NQO1, SOD, and CAT (Chen et al., 2010, Huang et al., 2024). Hesperidin also suppresses inflammatory signaling by inhibiting NF‑κB activation. This effect reduces the expression of downstream pro-oxidant enzymes such as iNOS and COX‑2, thereby limiting ROS production driven by inflammatory cascades (Chen et al., 2010, Sakata et al., 2003). The findings of the present study indicate that, beyond modulating oxidant–antioxidant balance, Hesperidin attenuated ethanol‑induced neuroinflammation in the hippocampus. This effect was evidenced by reduced expression of the inflammatory mediators GFAP and TNF‑α. Consistent with these observations, Yıldız et al. (2022) reported that Hesperidin significantly decreased cerebral levels of pro‑inflammatory cytokines, including IL‑1β, TNF‑α, and NF‑κB, in a sodium fluoride‑induced neurotoxicity model in rats. In the same study, Hesperidin reduced lipid peroxidation and enhanced antioxidant defenses by increasing SOD, CAT, and glutathione peroxidase (GPx) activities, as well as GSH levels in cerebral tissue (Yıldız et al., 2022). Similarly, Hesperidin treatment reduced IL‑1β and TNF‑α levels following spinal cord injury in rats. This intervention also increased total antioxidant status (TAS) while suppressing necrosis and apoptosis in spinal cord tissue (Yurtal et al., 2020). The neuroprotective properties of Hesperidin, manifested through its capacity to mitigate neuronal death and enhance neuronal survival, have been thoroughly documented in numerous scholarly investigations. Hesperidin possesses the ability to inhibit a wide array of cellular demise pathways, comprising apoptosis (Liu et al., 2017), necrosis (Yurtal et al., 2020), necroptosis (Wang et al., 2025), and autophagy (Huang et al., 2012), in addition to regulated modalities such as pyroptosis (Cao et al., 2023) and ferroptosis (Xin et al., 2025). Significantly, Hesperidin demonstrates modulatory influences on pivotal molecular regulators within these cell death pathways, as evidenced by existing literature with a specific focus on caspase-dependent apoptotic mechanisms (Hajialyani et al., 2019).

The findings of the present study demonstrate that the marked downregulation of caspase‑3 in the hippocampal CA1 region of FASD animals indicates that Hesperidin attenuates neuroapoptotic cell death. These results suggest that Hesperidin promotes neuronal survival in the developing hippocampus. These observations are consistent with previous reports from various neurotoxicity models. For example, Hesperidin showed strong anti‑apoptotic effects against methotrexate‑induced damage in hippocampal neural stem cells in Sprague‑Dawley rats. This protection was associated with reduced expression of Bax and caspase‑3 and increased levels of the pro‑survival protein Bcl‑2 (naewla et al., 2023). More broadly, numerous studies have documented the neuroprotective properties of Hesperidin, including its ability to reduce neuronal death, oxidative stress, and neuroinflammation in different experimental models (Hajialyani et al., 2019). These effects are frequently accompanied by improvements in cognitive and memory functions, as well as reduced anxiety‑like behaviors (Hajialyani et al., 2019, Kim et al., 2019). Despite this growing body of evidence, relatively few studies have examined the neurodevelopmental effects of Hesperidin, particularly its protective role in the developing brain. Consequently, this field remains insufficiently explored, and further research is needed to clarify the cellular, synaptic, and cognitive mechanisms underlying Hesperidin‑mediated neuroprotection during neurodevelopment (Kim et al., 2019). In this study, the prophylactic administration of Hesperidin was conducted in a neonatal Sprague–Dawley rat model exposed to sevoflurane anesthesia. The results revealed a significant decrease in the levels of apoptotic proteins within the hippocampus, including cleaved caspase−3, BAD, and BAX, in conjunction with a pronounced upregulation of pro-survival proteins Bcl−2 and Bcl-xL. Furthermore, Hesperidin suppressed neuroinflammatory processes by reducing the expression of major inflammatory cytokines, including TNF‑α, IL‑1β, and IL‑6. This anti‑inflammatory action was accompanied by reductions in ROS and MDA levels, along with increased SOD and CAT activities in hippocampal tissue, indicating a clear attenuation of sevoflurane‑induced oxidative stress following Hesperidin administration. Taken together, these antioxidant, anti‑oxidative‑stress, pro‑survival, and anti‑apoptotic properties, combined with Hesperidin’s strong anti‑inflammatory actions, resulted in marked improvements in hippocampus‑dependent behavioral performance. These enhancements included better learning and memory outcomes in the MWM, Y‑maze, and Fear‑Conditioning tests, as well as reduced anxiety‑like behaviors in the Open‑Field and EPM assessments (Kim et al., 2019).

## 5 Conclusion

Ethanol exposure during early postnatal development markedly induced anxiety-like behavior, impaired spatial learning and memory, elevated hippocampal MDA and TNF-α concentrations, and suppressed the antioxidant activities of SOD and GSH-Px. Moreover, ethanol significantly increased the number of GFAP- and caspase−3-positive cells within the CA1 region of the hippocampus, indicating pronounced astrogliosis and neuronal apoptosis. Hesperidin administration produced a dose-dependent restoration of behavioral performance in both the EPM and MWM tests, accompanied by normalization of oxidative and inflammatory markers and attenuation of GFAP and caspase−3 immunoreactivity. Collectively, these results demonstrate that hesperidin exerts substantive neuroprotective effects against ethanol-induced developmental neurotoxicity by reducing oxidative stress, suppressing neuroinflammation, and limiting apoptotic cell death in the hippocampal tissue.

## 6 Limitations

A notable limitation of the present study is its singular emphasis on male offspring (Mohseni and Rafaiee, 2026). Although male rodents were intentionally selected to reduce variability associated with hormonal changes during the estrous cycle (Inoue, 2022, Rocks et al., 2022) and in light of previous research suggesting a greater susceptibility of males to ethanol-induced neurotoxic effects during crucial stages of brain development (Alfonso-Loeches et al., 2013), this methodological decision limits the wider relevance of our findings. Female offspring might exhibit unique behavioral, neuroinflammatory, and oxidative stress responses to both ethanol exposure and Hesperidin treatment, potentially influenced by the regulatory effects of estrogen. Therefore, future investigations that include both sexes are essential to deepen our understanding of sex-specific vulnerability and the effectiveness of therapeutics in relation to developmental ethanol exposure (Miller et al., 2016).

## CRediT authorship contribution statement

**Fahimeh Mohseni:** Writing – review & editing, Validation, Supervision, Methodology, Investigation, Conceptualization. **Zhaleh Jamali:** Writing – original draft, Methodology, Investigation. **Behzad Garmabi:** Validation, Supervision, Project administration, Investigation. **Ahmad Salimi:** Writing – review & editing, Project administration, Methodology, Investigation. **Mehdi Khaksari:** Writing – review & editing, Supervision, Project administration, Methodology, Conceptualization.

## Ethics approval

this manuscript received approval from the Ethics Committee of Shahroud University of Medical Sciences, located in Iran. The ethics code assigned to this study is IR.SHMU.REC.1401.082. The identification number associated with the research project, designated as the tracking code, is 140091.

## Funding

This research endeavor was financially supported by Grant No. 140091, conferred by 10.13039/501100004305Shahroud University of Medical Sciences.

## Conflicts of Interest

The authors declare that there are no conflicts of interest regarding the publication of this article. All experimental procedures involving animals were conducted in accordance with the ethical standards and guidelines for the care and use of laboratory animals and were approved by the Ethics Committee of Shahroud University of Medical Sciences, Shahroud, Iran (Ethics code: IR.SHMU.REC.1401.082; Project tracking code: 140091).

## Data Availability

Data will be made available on request.
